# All purulence is local – epidemiology and management of skin and soft tissue infections in three urban emergency departments

**DOI:** 10.1186/1471-227X-13-26

**Published:** 2013-12-20

**Authors:** Chris Merritt, John P Haran, Jacob Mintzer, Joseph Stricker, Roland C Merchant

**Affiliations:** 1 Department of Emergency Medicine, Alpert Medical School of Brown University and Rhode Island Hospital/Hasbro Children’s Hospital, Providence, RI, USA; 2 Department of Emergency Medicine, University of Massachusetts Medical School and UMass Memorial Medical Center, Worcester, MA, USA

**Keywords:** Skin and soft tissue infections, Antibiotic resistance, Antimicrobial stewardship

## Abstract

**Background:**

Skin and soft tissue infection (SSTIs) are commonly treated in emergency departments (EDs). While the precise role of antibiotics in treating SSTIs remains unclear, most SSTI patients receive empiric antibiotics, often targeted toward methicillin-resistant *Staphylococcus aureus* (MRSA). The goal of this study was to assess the efficiency with which ED clinicians targeted empiric therapy against MRSA, and to identify factors that may allow ED clinicians to safely target antibiotic use.

**Methods:**

We performed a retrospective analysis of patient visits for community-acquired SSTIs to three urban, academic EDs in one northeastern US city during the first quarter of 2010. We examined microbiologic patterns among cultured SSTIs, and relationships between clinical and demographic factors and management of SSTIs.

**Results:**

Antibiotics were prescribed to 86.1% of all patients. Though *S. aureus* (60% MRSA) was the most common pathogen cultured, antibiotic susceptibility differed between adult and pediatric patients. Susceptibility of *S. aureus* from ED SSTIs differed from published local antibiograms, with greater trimethoprim resistance and less fluoroquinolone resistance than seen in *S. aureus* from all hospital sources. Empiric antibiotics covered the resultant pathogen in 85.3% of cases, though coverage was frequently broader than necessary.

**Conclusions:**

Though *S. aureus* remained the predominant pathogen in community-acquired SSTIs, ED clinicians did not accurately target therapy toward the causative pathogen. Incomplete local epidemiologic data may contribute to this degree of discordance. Future efforts should seek to identify when antibiotic use can be narrowed or withheld. Local, disease-specific antibiotic resistance patterns should be publicized with the goal of improving antibiotic stewardship.

## Background

Emergency department (ED) visits in the US for skin and soft-tissue infections (SSTIs) have more than tripled in number in recent decades, [1,2] mirroring the emergence of community-acquired methicillin-resistant *Staphylococcus aureus* (CA-MRSA) [3,4]. Two types of bacterial SSTIs predominate among ED patients: cellulitis, typically a non-purulent bacterial skin infection; and abscesses, characterized by collections of purulent fluid. Though the current epidemiology of cellulitis is understudied, the most common circulating strains of CA-MRSA have a well-described predilection for causing abscesses, and are the primary pathogens in these purulent SSTIs in many areas [5].

Prevalence of CA-MRSA varies from region to region. Most hospitals publish antibiotic susceptibility data from their own microbiology laboratories. Commonly called “antibiograms”, these documents are important tools for use by front-line clinicians in making educated treatment decisions. However, they typically report aggregate data based on bacterial isolates from all sources (blood, skin, sputum, etc.), and infrequently delineate pathogens based on the age of the patient or the source of the infection.

Although healthcare exposure appears to remain a risk factor for drug-resistant infections, ED clinicians are left with few additional demographic or clinical clues to the likelihood of resistant organisms in SSTI patients without exposures. Investigators have also noted differences in microbiology and treatment of pediatric and adult SSTIs [6]. Children beyond the neonatal period have been considered high-risk for CA-MRSA SSTIs relative to adults, though as the CA-MRSA epidemic has matured, this distinction has become less clear [7].

Current guidelines for treatment of CA-MRSA infections do not call for routine antibiotics for adequately drained, uncomplicated abscesses [8]. Nonetheless, while incision and drainage (I&D) remains the primary treatment for abscesses, clinicians prescribe antibiotics for the majority of these patients and empiric prescription of antibiotics typically active against CA-MRSA has become routine [9-13]. In addition, many clinicians provide “double coverage”, which we define as using two or more antibiotics with the intention of effectively treating MRSA, methicillin-sensitive *S. aureus* (MSSA) and β-hemolytic *Streptococcus*[14,15].

Because antibiotics increase the cost of treatment, the incidence of adverse medication effects, and – importantly – the selective pressure leading to further antibiotic resistance, their precise role continues to be debated [16-21]. Given the inability to predict resistance based on clinical factors, some discordance between empiric treatment and pathogen is inevitable. Factors related to this discordance have not been well studied. If antibiotic choices are not well targeted, ED patients with purulent SSTIs may represent a population in whom antibiotic use could effectively be reduced, decreasing the selective pressures, cost burdens, and unintended side effects of these medications.

In this study, our primary objective was to assess – based on ED antibiotic prescribing choices and culture results – the efficiency with which ED clinicians targeted specific pathogens, particularly CA-MRSA, with empiric antibiotics. Focusing on infections that were most likely to be community-acquired rather than healthcare-associated, we assessed whether patient demographics and clinical features of presumed community-acquired SSTIs might have led emergency clinicians to prescribe empiric antibiotic therapy discordant with the susceptibility of the cultured pathogen or to institute multi-drug “double coverage”. Because epidemiology and practice patterns are likely to differ in pediatric and adult patients, we examined management differences between children and adults in the ED with presumed-community-acquired SSTIs. Additionally we sought to determine the prevalent local microbiologic and practice patterns in ED patients treated for SSTIs.

## Methods

### Study design and setting

A retrospective analysis of patient visits for suspected community-acquired SSTIs to three urban, academic EDs located in one New England city was performed for the first quarter of 2010 (January 1 through March 31). The EDs included an urban adult ED in a large academic hospital, a pediatric ED in the affiliated academic children’s hospital, and an academically-affiliated community hospital. Together, the three EDs care for approximately 200,000 patients per year. This study was approved by the institutional review board of the Lifespan Corporation and was performed in accordance with the appropriate guidelines for protection of human subjects and protected health information.

### Study population

Two billing databases containing data for all patient visits to each of the study EDs – one from the hospitals’ billing system and one from the physician practice that staffs the EDs – were combined to maximize catchment. ED visits for all patients diagnosed with SSTIs were identified from the combined database using International Classification of Diseases, 9^th^ Edition (ICD-9) diagnosis codes 680–686 (Infection of Skin and Subcutaneous Tissue). Duplicate records due to the combination of datasets were eliminated. Repeat visits to the ED for the same SSTI by the same patient also were eliminated from the study, as were patients deemed to have likely healthcare-associated infection by virtue of having been hospitalized or having surgery within the previous 3 months, or currently residing in a skilled nursing facility. The latter were identified by record review from the study hospitals, mention in the physician chart, or identifying the patient’s address at a skilled nursing facility.

### Study protocol

The protocol adhered to recommendations on the optimal conduct of retrospective studies for emergency medicine [22]. A research assistant (RA), blinded to the study objectives, reviewed and abstracted data from the electronic medical record into a data collection form, recording demographic, historical and clinical data, and the clinician’s diagnosis as recorded by the treating clinician (irrespective of ICD-9 code assigned by billers). A second RA, blinded to clinical and historical data and using the hospital’s microbiology laboratory reporting record, recorded whether or not a culture was ordered in the ED and recorded the resulting isolates’ antimicrobial susceptibilities. RAs were trained by the primary investigator (PI), who met regularly with RAs for monitoring of case selection and data management.

ED visits identified by ICD-9 code that were in fact not for SSTIs (i.e. coding errors) were excluded following verification by the PI. The PI reviewed ten percent of records for data quality and to assess interrater reliability on three key variables. The kappa statistic for performance of culture in the ED was 0.81, for performance of I&D was 0.79, and for infection type was 0.90.

### Outcome measures

Descriptive measures included MRSA prevalence and antibiotic prescribing patterns among cultured SSTIs in the study ED populations. Primary outcomes measured were (a) the frequency of in vitro activity of ED clinicians’ empiric antibiotic therapy against the cultured isolates among ED patients with cultured SSTIs, (b) factors associated with use of discordant antibiotic therapy or multi-drug antibiotic therapy, and (c) antibiotic resistance patterns among the most common pathogens identified.

### Data analysis

We estimated that 25% of all SSTI patients evaluated in the ED would undergo culture and that 90% of these patients would receive antibiotics, with 50% concordance for MRSA treatment when MRSA was isolated. Given these assumptions, between 674 and 1199 patient records would need to be abstracted to arrive at an estimate of antibiotic/culture discordance with 95% confidence intervals encompassing a range of 15 to 20 percentage points.

Antibiotic usage was stratified by culture results, and age groups were compared using Pearson’s chi-square and 2-sample tests of binomial proportions.

Antibiotics were categorized based on their spectrum of activity. Anti-staphylococcal antibiotics typically active against CA-MRSA include trimethoprim-sulfamethoxazole, tetracycline, doxycycline, clindamycin, rifampin, linezolid or vancomycin [1,2,13]. Antibiotics with anti-staphylococcal properties but typically ineffective against CA-MRSA were categorized as “MSSA antibiotics”: penicillins, first-generation cephalosporins, macrolides, and fluoroquinolones. “Double coverage” describes treatment with two or more antibiotics with gram-positive coverage. Three univariable logistic models were created to identify demographic or clinical variables associated with (1) *in vitro* coverage of any organism isolated by the empiric ED antibiotic therapy, (2) use of double antibiotic coverage, and (3) discordance between treatment and culture. In the third regression model, concordance was defined as presence of MRSA in culture when any anti-MRSA treatment was prescribed or presence of MSSA in culture when only anti-MSSA treatment was prescribed. Discordance is the converse. Data analysis was performed using Stata (StataCorp, College Station, TX).

## Results

### The study population

The ICD-9 code search yielded 1,158 separate ED visits for SSTI, of which 1,094 (94.5%) were initial visits for SSTIs. The remaining 64 ED visits constituted either return visits for the same infection or ICD-9 mis-coding. Of the 1,094 ED visits, 160 (14.6%) represented patients with known healthcare exposure, leaving 936 patients – the study population – in whom the SSTI was likely community-acquired.

Table 1 summarizes demographic and clinical characteristics of the study population, stratified by age group. As compared to adult community-acquired SSTI patients, pediatric patients were more likely to be female, non-white, and insured. In addition, pediatric SSTI patients were more likely to have a diagnosis other than abscess or cellulitis (primarily impetigo or paronychia, data not shown). As compared to adults, more pediatric abscesses occurred on the buttock (28.8% vs. 15.4%; p < 0.05) and fewer on the face (6.9% vs. 15.8%; p < 0.05).

**Table 1:** Demographic and clinical characteristics of ED patients with community-acquired Skin and Soft-tissue infections (SSTIs) by age group

***Demographic and clinical characteristics***	**Pediatric (age < 18)**	**Adult (age ≥18)**	**p-value**
	***n = 191***	***n = 745***	
	**%**	**%**	**p<**
**Median age in years (IQR)**	**7 (0–17)**	**39 (18–89)**	***p < 0.001***
**Gender**			***p < 0.02***
Male	**47.6**	**57.2**	
Female	**52.4**	**42.8**	
**Ethnicity/race**			***p < 0.001***
White	**52.9**	**67.9**	
Black	**22.0**	**17.6**	
Hispanic/Other	**25.1**	**14.5**	
**Health insurance status**			***p < 0.001***
Private	**36.6**	**33.4**	
Governmental	**44.5**	**31.3**	
Both Private and Governmental	**8.9**	**7.0**	
Uninsured	**10.0**	**28.3**	
**Infection type**			**p < 0.001**
Abscess	**38.3**	**41.9**	
Cellulitis	**44.0**	**51.0**	
Furuncle	**0.5**	**0.5**	
Carbuncle	**0.5**	**0.0**	
Ulcer	**0.5**	**0.3**	
Other	**16.2**	**6.3**	
**Previous assessment for this infection**	**28.3**	**25.8**	**p < 0.5**
**Previous antibiotic treatment for this infection**	**20.9**	**18.2**	**p < 0.4**

IQR = interquartile range.

### ED management of suspected community-acquired SSTIs

Among suspected community-acquired SSTIs, of the ED patients diagnosed with abscesses, pediatric and adult patients were equally likely to undergo I&D in the ED (58.9% and 65.6%; p < 0.29), but microbiologic culture was ordered more often in the pediatric patients (65.8% vs. 47.6%; p < 0.005).

The majority of patients with suspected community-acquired SSTIs were evaluated in the ED and discharged. Pediatric patients with abscesses were more likely than adults with abscesses to be admitted to hospital (34.3% vs. 14.5%; p < 0.001).

### Antibiotic use

Antibiotics (whether intravenous (IV) or oral, used in the ED or prescribed at discharge, or any combination of these) were prescribed to 86.1% of the 936 ED patients with suspected community-acquired SSTIs (94% of those with cellulitis vs. 78.4% of those with abscess; p < 0.0001). For patients with cellulitis, 93.9% of adult and 94.1% of pediatric patients were prescribed antibiotics (p < 0.97); for those with an abscess, 76.9% of adult and 84.9% of pediatric patients were prescribed antibiotics (p < 0.14); and for all other suspected community-acquired SSTIs, 73.6% of adult and 85.3% of pediatric patients were prescribed antibiotics (p < 0.20).

Overall, 38.2% of SSTI patients (88.6% of admitted patients and 15.7% of discharged patients) received IV antibiotics in the ED, more frequently in adults than in children (40.4% vs. 29.8%; p < 0.009). The most commonly prescribed IV antibiotics for adults were vancomycin (24.9%), ampicillin/sulbactam (11.4%), and, cefazolin (7.9%), and for children were clindamycin (15.7%), cefazolin (5.8%), and ampicillin/sulbactam (4.7%). Adult patients were more likely than pediatric patients to receive IV vancomycin (24.9 vs. 1.6%; p < 0.001) and ampicillin/sulbactam (11.4% vs. 4.7%; p < 0.007), but were less likely than pediatric patients to receive IV clindamycin (4.6% vs. 15.7%; p < 0.001).

Among discharged patients, trimethoprim-sulfamethoxazole (TMP-SMX), clindamycin, and cephalexin were prescribed commonly. 25.3% of all SSTI patients received an oral antibiotic in the ED, and 80.8% of patients discharged received an antibiotic prescription. Adult SSTI patients were more likely than pediatric SSTI patients to be prescribed oral TMP-SMX (59.1% vs. 37.2%; p < 0.001) at ED discharge. Of those prescribed TMP-SMX, adults were more likely than children to be also prescribed oral cephalexin (53.8% vs. 34.5%; p < 0.001).

### Microbiologic data

Table 2 summarizes the results of microbiologic cultures by age group among ED patients with cultured infections. *S. aureus* comprised the majority of culture isolates, and was more common in pediatric patients, while mixed flora was more common among adults than among children. Of *S. aureus* isolated from CA-SSTIs, 60.4% was categorized as MRSA, with similar proportions in adults and children (60.9% vs. 59.2%; p < 0.84).

**Table 2:** Microbiologic culture results from emergency department (ED) patients with community-acquired skin & soft tissue infections

**Culture result**	**Pediatric n = 70% (% pre-treated)**	**Adult n = 241% (% pre-treated)**
No Growth	7.4	9.6
	(80)	(25)
*Staphylococcus aureus*	72.1	52.6
	(20.4)	(21.8)
MRSA	44.3	31.1
MSSA	27.8	21.5
Group B *Streptococcus*	1.5	3.8
	(0)	(12.5)
Group A *Streptococcus*	7.4	1.4
	(0)	(0)
*Escherichia coli*	0	1.9
		(0)
*Pseudomonas aeruginosa*	0	1.4
		(0)
*Klebsiella* species	0	0.5
		(0)
Mixed Skin Flora	3	16.8
	(0)	(11.4)
Other Mixed Flora^*a*^	0	2.4
		(20)
Other	8.6	9.6
	(33.3)	(15)

^*a*^Includes Mixed Oral Flora and Mixed GI Flora.

%Pre-treated depicts the percentage of patients identified as having received prior antibiotic treatment for the infection in question.

MRSA = methicillin-resistant *S. aureus*, MSSA = methicillin-sensitive *S. aureus.*

Table 3 depicts the antibiotic susceptibilities among *S. aureus* isolated from presumed community-acquired purulent infections, comparing the resistance in these ED-acquired SSTI cultures against the resistance reported for MRSA and MSSA on the antibiogram distributed by the hospitals’ microbiology laboratory for *S. aureus* from all sources in 2010.

**Table 3:** **Antibiotic Resistance Among *Staphylococcus aureus *** **Isolated from skin and soft tissue infections (SSTIs) in the emergency department**

	**MSSA**			**MRSA**		
	**ED Samples (n = 76; 56 Adult)**		**Antibiogram (n = 619)**	**ED Samples (n = 101; 70 Adult)**		**Antibiogram (n = 473)**
**Drug**	**Pediatric% resistant**	**Adult% resistant**	**Total% resistant**	**Pediatric% resistant**	**Adult% resistant**	**Total% resistant**
Ciprofloxacin	0%	2.9%	14%	19.3%	24.5%	61%
Clindamycin	0%	14.7	NR	9.7%	4.1%	NR
Erythromycin	**28.6%**	**41.2%**	39%	**87.1%**	**100%**	92%
Gentamicin	0%	0%	0%	0%	0%	1%
Levofloxacin	0%	2.9%	13%	19.3%	22.5%	60%
Moxifloxacin	0%	2.94%	NR	NR	NR	NR
Oxacillin^*a*^	0%	0%	0%	100%	100%	100%
Penicillin	85.7%	82.3%	NR	100%	100%	NR
Tetracycline	14.3%	5.9%	5%	0%	0%	2%
TMP/SMX	**35.7%**	**2.9%**	1%	0%	0%	1%
Vancomycin	0%	0%	0%	0%	0%	0%
Inducible Clindamycin Resistance	**0%**	**14.7%**	NR	3.2%	4.1%	NR

Bolded pairs achieved statistical significance (p < 0.05) in chi-square analysis.

MSSA = methicillin-sensitive *S. aureus*; MRSA = methicillin-resistant *S. aureus*; TMP/SMX = Trimethoprim/Sulfamethoxazole; NR = Not Reported

^*a*^Oxacillin resistance defines MRSA vs. MSSA in this clinical laboratory.

### Discordance of antibiotic therapy with culture results

Table 4 compares antibiotic treatment with culture results among SSTIs for which both antibiotics were prescribed and cultures obtained. Clinicians using single antibiotics (anti-MRSA [Table 4A] or anti-MSSA [Table 4B]) used monotherapy that accurately targeted the resultant cultured pathogen accurately 39.3% of the time (ranging from 35-52% depending on age and treatment strategy). Cultured SSTIs from 100% of pediatric patients and 67.8% of adult patients treated with multi-drug “double coverage” (Table 4C) grew only *Staphylococcus*.

**Table 4:** Empiric antibiotic treatment and targeted organisms in microbiologic culture

**A.**	**Culture results**					
	**Pediatric**		**Adult**			
	**MRSA%**	**Other result%**	**MRSA%**	**Other result%**		
Anti-MRSA therapy only	52.6	47.4	34.5	65.5		
**B**						
	**Culture results**					
	**Pediatric**		**Adult**			
	**MSSA%**	**Other result%**	**MSSA%**	**Other result%**		
Anti-MSSA therapy only	37.5	72.5	35	65		
**C.**						
	**Culture results**					
	**Pediatric**			**Adult**		
	**MRSA%**	**MSSA%**	**Other result%**	**MRSA%**	**MSSA%**	**Other result%**
Double Coverage	62.5	37.5	0.0	44.3	21.5	34.2

MSSA = methicillin-sensitive *S. aureus*; MRSA = methicillin-resistant *S. aureus.*

*NB :* Patients (63 pediatric, 189 adult) with skin and soft tissue infections (SSTIs) that were cultured in the ED and who received antibiotics are shown, with pediatric and adult patients considered separately. Tables are separated by empiric therapy given and the culture result targeted. 4A: Patients who were treated with empiric anti-MRSA antibiotics only. 4B: Patients who were treated with empiric anti-MSSA antibiotics only. 4C: Patients who were treated with double coverage (treatment with two or more antibiotics with gram-positive coverage).

Anti-MRSA antibiotics include antibiotics typically active against community-acquired (CA-) MRSA: trimethoprim-sulfamethoxazole, tetracycline, doxycycline, clindamycin, rifampin, linezolid or vancomycin. Anti-MSSA antibiotics include anti-staphylococcal antibiotics typically ineffective against CA-MRSA (penicillins, cephalosporins, macrolides, fluoroquinolones).

Table 5 displays the univariable logistic regression analyses investigating demographic and clinical correlates of coverage of the resultant pathogen with the chosen antibiotic regimen, “double coverage” antibiotic usage, and discordance of empiric MRSA therapy (use of anti-MRSA antibiotics in the absence of MRSA, or vice versa).

**Table 5:** Logistic models to identify factors associated by univariate analysis with coverage of the isolated organism by the antibiotic prescribed (A), treatment with multiple antibiotics (“double coverage”) (B), and discordant anti-mrsa therapy (C)

**Variable**	**(A) Coverage of the organism isolated**^**Δ **^**(n=156) or (95%C.I.)**	**(B) Double coverage (n = 804) or (95%C.I.)**	**(C) Discordant anti-mrsa therapy (n = 181) or (95%C.I.)**
**Age group (adult vs. pediatric)**	**1.92 (0.78-4.76)**	**5.60 (3.39-9.25)**	**1.63 (0.82-3.23)**
**Infection Location**			
Hand	**Ref.**	**Ref.**	**Ref.**
Other extremity	**1.71 (0.45-6.44)**	**1.37 (0.87-2.18)**	**1.16 (0.41-3.25)**
Buttock	***	**1.07 (0.55-2.11)**	**1.00 (0.28-3.54)**
Trunk	**2.31 (0.5-10.67)**	**1.00 (0.56-1.78)**	**0.64 (0.22-1.87)**
Head	**0.62 (0.1-3.66)**	**1.15 (0.44-2.96)**	**4.00 (0.40-39.83)**
Face	**4.92 (0.49-49.61)**	**0.95 (0.54-1.68)**	**0.46 (0.13-1.59)**
Genitalia	**0.31 (0.02-6.11)**	**1.07 (0.30-3.81)**	**0.67 (0.04-12.27)**
Multiple Locations	**1.23 (0.11-14.42)**	**1.07 (0.45-2.54)**	**0.67 (0.08-5.75)**
**Previous assessment for this infection**	**1.27 (0.4-4.02)**	**0.85 (0.60-1.20)**	**0.86 (0.42-1.79)**
**Previous antibiotic treatment for this infection**	**4.13 (0.53-32.28)**	**0.81 (0.54-1.22)**	**0.45 (0.19-1.07)**
**Disposition (hospital admission)**	2.78 (1.12-6.89)*	**0.99 (0.73-1.35)**	**0.66 (0.33-1.32)**
**I&D performed**	4.47 (1.71-11.65)*	**1.07 (0.77-1.47)**	**0.87 (0.47-1.60)**
**Culture performed**	**N/A**	**1.37 (1.00-1.87)**	**N/A**
**Gender**			
Male	**Ref.**	**Ref.**	**Ref.**
Female	**0.92 (0.38-2.24)**	**0.75 (0.56-1.00)**	**0.82 (0.45-1.47)**
**Ethnicity/race**			
White	**Ref.**	**Ref.**	**Ref.**
Black	**1.58 (0.49-5.05)**	0.65 (0.43-0.97)*	**0.89 (0.41-1.89)**
Hispanic/Other	**5.62 (0.71-44.27)**	**0.72 (0.48-1.09)**	**1.06 (0.46-2.42)**
**Health insurance status**			
Private	**Ref.**	**Ref.**	**Ref.**
Governmental	**1.31 (0.41-4.19)**	**0.94 (0.66-1.34)**	**0.85 (0.40-1.77)**
Both private and Governmental	0.11 (0.02-0.5)*	**0.80 (0.45-1.43)**	**1.94 (0.36-10.50)**
None	**1.63 (0.44-5.98)**	**1.23 (0.84-1.81)**	**0.69 (0.33-1.47)**

^**Δ**^excludes cultures with no growth or mixed flora and patients not treated with antibiotics.

* denotes statistically significant result.

*** variable had perfect prediction in the model.

NB: Three univariable logistic models were created to identify demographic or clinical variables associated with (A) coverage of the organism isolated by the empiric ED antibiotic therapy, (B) use of multiple antibiotics as empiric therapy (“double coverage”), and (C) discordance between use of anti-MRSA antibiotics and presence of MRSA as identified by microbiologic culture. In the third regression model, concordance was defined as presence of MRSA in culture when *any* anti-MRSA treatment was prescribed or presence of MSSA in culture when *only* anti-MSSA treatment was prescribed.

Patients who underwent I&D were more likely to receive antibiotics that covered the resultant pathogen than those who did not undergo the same procedure in the ED. Patients admitted to the hospital were more likely to receive antibiotics in the ED to which the resultant pathogen was susceptible than those discharged home.

Age group was strongly associated with treatment with two or more antistaphylococcal antibiotics, with adult patients more likely than pediatric patients to receive such multiple antibiotic coverage. Black patients were less likely than non-black patients to receive multi-drug coverage. However, when age and race were considered jointly as correlates, only adult age remained associated with greater “double coverage” usage.

There were no demographic or clinical factors identified in association with discordance between presence or absence of empiric anti-MRSA antibiotic therapy and the presence or absence of MRSA among those undergoing culture and receiving antibiotics.

## Discussion

Emergency clinicians routinely make decisions for SSTIs based on incomplete information; treatment guidelines remain vague regarding when antibiotics are indicated, information about local epidemiology is often incomplete, and microbiologic data for individual patients are not available in the time frame of an ED visit.

In this study, we identified a population of ED patients with presumed community-acquired SSTIs in whom *S. aureus* remained the most common pathogen and for whom antibiotic prescription remained high. Despite the prevalence of *S. aureus* as the target of therapy, antibiotic regimens varied significantly. Among patients who underwent culture and received antibiotics, discordance between the choice to treat empirically with anti-MRSA antibiotics and the presence or absence of the resistant organism in culture was high; patients were often treated narrowly for MRSA infections, or broadly for MSSA infections.

The microbiology of skin abscesses does not appear to be uniform; resistance patterns from our sample differed between children and adults. Increased resistance to TMP/SMX – among the most commonly-used antibiotics in SSTIs – was noted, particularly in MSSA isolated from children. Though the number of pediatric MSSA infections was a small proportion of the total number of patients, 20 of the 49 *S. aureus* cultures from children were MSSA. The implications of this finding are not immediately clear, but highlight the importance of (a) knowledge of local disease epidemiology, and (b) performance of surveillance cultures in at least some subset of ED patients treated for SSTIs. This epidemiologic surveillance is important in monitoring infections treated in the ED, and may identify emerging resistance before it becomes broadly apparent.

Importantly, differences in disease epidemiology were not reflected in the antibiogram distributed by the hospitals’ microbiology laboratory. *S. aureus* from SSTIs had greater TMP/SMX resistance and less fluoroquinolone resistance than reflected in the antibiogram. The resistance patterns reflected in composite antibiograms may mask important differences in pathogens’ behavior in SSTIs, since the antibiogram does not distinguish between pathogens isolated from blood, sputum, or other sources. The strains of *S. aureus* that cause purulent SSTIs differ from those that cause other invasive infections, and this may not be clear when microbiologic data is viewed in the aggregate. Laboratories should consider reporting disease-specific antibiotic resistance data, as this more granular data could drive therapeutic decision-making.

None of the demographic or clinical factors in our logistic model correlated choice of empiric anti-MRSA therapy with the presence or absence of MRSA in culture in our sample of patients who underwent culture and received antibiotics. Using prescribing behavior as a proxy for clinician beliefs, there did not appear to be specific factors interpreted by ED clinicians as being predictive of a particular pathogen’s antibiotic susceptibility. However, those patients who were admitted to the hospital or who underwent I&D in the ED were more likely to receive antibiotic therapy in the ED to which the resultant cultured organism was susceptible, suggesting that those patients deemed to be more ill or to require an invasive procedure were more likely to receive broader antibiotic therapy.

Use of “double coverage” – two or more antibiotics, typically TMP-SMX plus cephalexin – was prevalent, and was likely intended to address perceived deficiencies of single-agent treatment with TMP-SMX in treating streptococci. However, cultures from the large majority of patients treated with “double coverage” yielded staphylococci alone, suggesting that empiric anti-streptococcal treatment may not be necessary. When viewed from an antibiotic stewardship perspective, “double coverage” doubles the exposure to antibiotics and may drive resistance without leading to improved therapy.

Only age group was reliably associated with use of “double coverage” in our logistic model; children were less likely to receive multiple antibiotics. Otherwise, the choice appears to be one of clinician discretion. Given that most isolates even from adult patients yielded staphylococci, and that I&D alone is sufficient therapy for most uncomplicated abscesses, use of a single antibiotic – chosen using local epidemiologic data, where available – is warranted if antibiotics are deemed necessary.

The clinician can opt not to treat uncomplicated, small purulent infections with antibiotics if adequate I&D is performed. This is increasingly supported by the evidence and in recent guidelines for treatment of CA-MRSA infections, and is not likely to decrease treatment failure or increase selective pressures toward antibiotic resistance [8].

We acknowledge several limitations to the current study. Its retrospective nature limits the data to that which could be collected from the medical record. However, we believe that we have adhered to high standards for retrospective ED studies [22,23]. We could not directly assess the ED clinicians’ intention when choosing which SSTIs to treat with which antibiotics, and could only infer from those choices. Medical records rarely described SSTIs in detail, omitting the degree of cellulitis adjacent to an abscess. We attempt to account for this by limiting our analysis to the “accuracy” of antibiotic choices without inferring the clinicians’ specific intent.

Our findings are significant in that they reflect the current state of antibiotic use and overuse. We were unable to correlate the choices of empiric antibiotics provided in the study EDs with any of the demographic or clinical variables studied. Clinicians, given the state of epidemiologic data and clinical tools available during the study period, had insufficient information to predict the susceptibility of an SSTI pathogen at the time that empiric therapy was chosen. If a clinician could (a) determine which purulent SSTIs require antibiotic treatment, and (b) estimate the narrowest effective antibiotic coverage using local disease-specific data or other tools, antibiotic overuse could be limited.

Future efforts in ED management of purulent SSTIs may focus on determining which patients benefit from antibiotic therapy, outcomes in patients treated without antibiotics, and ensuring that adequate I&D can be performed in the ED setting. PCR and other rapid-MRSA-testing technologies are becoming widely available, [24] though these newer technologies have not yet been widely studied in the clinical setting.

## Conclusion

*Staphylococcus aureus* is the predominant pathogen in community-acquired purulent SSTIs in the ED, and most patients evaluated for these infections received antibiotics even after I&D. Although antibiotic use, including multi-drug “double coverage”, remained common in the sample studied, empiric antibiotics used varied widely, and were poorly targeted toward the causative organisms, all of which represents an opportunity to reduce antibiotic overuse. Local epidemiologic data is critical to the decision-making of ED clinicians, and laboratories should consider reporting disease-specific antibiograms. Future efforts to identify SSTIs in which antibiotic use, particularly anti-MRSA therapy, is indicated could further reduce antibiotic overuse and improve antibiotic stewardship.

## Competing interests

The authors declare that they have no competing interests.

## Authors’ contributions

CM conceived of the study, and participated in and oversaw its design and coordination. JPH participated in data collection and made significant contributions to analysis and manuscript review. JM and JS were instrumental in design of data collection instruments and data abstraction and data management. RCM participated in design and conception of the study, and provided guidance throughout its course, from conception through manuscript review. All authors read and approved the final manuscript.

## Pre-publication history

The pre-publication history for this paper can be accessed here:

http://www.biomedcentral.com/1471-227X/13/26/prepub
